# 1,2-Diphenyl-2-(*m*-tolyl­amino)ethanone^1^

**DOI:** 10.1107/S1600536810013371

**Published:** 2010-04-17

**Authors:** Rafael Mendoza Meroño, Felix Nápoles Esculary, Laura Menéndez Taboada, Santiago García-Granda

**Affiliations:** a Departamento de Química Física y Analítica, Facultad de Química, Universidad de Oviedo, C/ Julián Clavería, 8, 33006 Oviedo, Spain; b Departamento de Química, Facultad de Ciencias Naturales, Universidad de Oriente, Santiago de Cuba, Cuba

## Abstract

The title compound, C_21_H_19_NO, belongs to the family of α-amino­ketones. The structure contains three benzene rings, two of which [the phenyl ring in the 1-position (*B*) and the methylaniline ring (*A*)] are nearly coplanar [dihedral angle = 5.4 (1)°], whereas the phenyl ring in the 2-position (*C*) is nearly normal to them [dihedral angles = 81.8 (1) and 87.0 (1)° for *A*/*C* and *B*/*C*, respectively]. The conformation of the N—H bond is *syn* to the C=O bond, favouring the formation of a centrosymmetric dimer of mol­ecules in the crystal structure. The mol­ecular packing is consolidated by this N—H⋯O hydrogen-bonding network.

## Related literature

For the structure of alpha-amino­ketones, see: Batsanov *et al.* (2006 ▶). For the crystal structure of 1,2-diphenyl-2-(*p*-tolyl­amino)ethanone, see: Au & Tafeenko (1986 ▶).
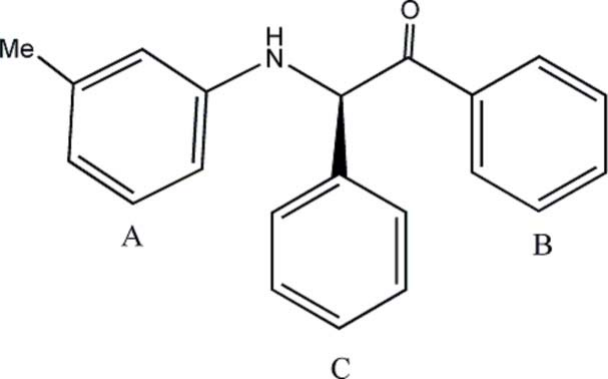


## Experimental

### 

#### Crystal data

C_21_H_19_NO
*M* *_r_* = 301.37
Triclinic, 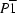

*a* = 6.0510 (3) Å
*b* = 11.5745 (4) Å
*c* = 12.9458 (7) Å
α = 112.542 (5)°
β = 97.396 (4)°
γ = 99.960 (4)°
*V* = 805.62 (8) Å^3^
*Z* = 2
Cu *K*α radiation
μ = 0.59 mm^−1^
*T* = 293 K
0.34 × 0.12 × 0.07 mm

#### Data collection

Oxford Diffraction Xcalibur Gemini S diffractometer
Absorption correction: refined from Δ*F* [cubic fit to sin(theta)/lambda - 24 parameters; Parkin *et al.* (1995 ▶)] *T*_min_ = 0.919, *T*_max_ = 0.960
8027 measured reflections
2833 independent reflections
2174 reflections with *I* > 2σ(*I*)
*R*_int_ = 0.027

#### Refinement

*R*[*F* ^2^ > 2σ(*F* ^2^)] = 0.043
*wR*(*F* ^2^) = 0.137
*S* = 1.09
2833 reflections
213 parameters
H atoms treated by a mixture of independent and constrained refinement
Δρ_max_ = 0.25 e Å^−3^
Δρ_min_ = −0.16 e Å^−3^

### 

Data collection: *CrysAlis CCD* (Oxford Diffraction, 2008 ▶); cell refinement: *CrysAlis RED* (Oxford Diffraction, 2008 ▶); data reduction: *CrysAlis RED*; program(s) used to solve structure: *SIR92* (Altomare *et al.*, 1994 ▶); program(s) used to refine structure: *SHELXL97* (Sheldrick, 2008 ▶); molecular graphics: *ORTEP-3 for Windows* (Farrugia, 1997 ▶); software used to prepare material for publication: *WinGX* (Farrugia, 1999 ▶) and *PLATON* (Spek, 2009 ▶).

## Supplementary Material

Crystal structure: contains datablocks global, I. DOI: 10.1107/S1600536810013371/su2170sup1.cif

Structure factors: contains datablocks I. DOI: 10.1107/S1600536810013371/su2170Isup2.hkl

Additional supplementary materials:  crystallographic information; 3D view; checkCIF report

**Fig. 1.**
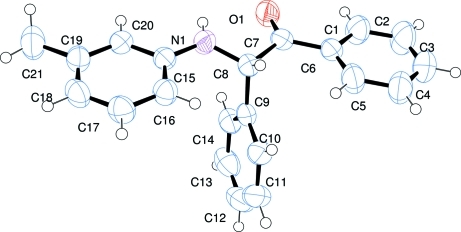
A view of the molecular structure of the title molecule showing the atom-numbering scheme. Displacement ellipsoids are drawn at the 50% probability level.

Enhanced figure: interactive version of Fig. 1

## Figures and Tables

**Table 1:** Hydrogen-bond geometry (Å, °)

*D*—H⋯*A*	*D*—H	H⋯*A*	*D*⋯*A*	*D*—H⋯*A*
N1—H22⋯O1^i^	0.859 (17)	2.660 (17)	3.3913 (17)	143.8 (15)

Symmetry code: (i) 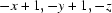.

**Fig. 2.**
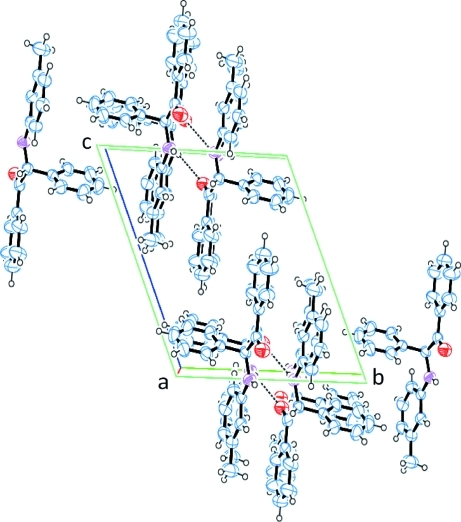
A view along the a-axis of the crystal packing of the title compound. Hydrogen bonds are indicated by dashed lines (see Table 1 for details).

## supplementary crystallographic information

### Comment

The structure of various members of the alpha-aminoketone family have been extensively studied (Batsanov *et al.*, 2006). These compounds can be used as intermediates to synthesize other biologically active compounds like thiosemicarbazones. Alpha-aminoketones also exhibit biological activity but are less active than the thiosemicarbazones. They are generally synthesised by the reaction of an alpha-hydroxiketone with an amine.

The molecular structure of the title molecule is illustrated in Fig. 1. According to the dihedral angles between the benzene rings planes, two benzene rings are nearly coplanar whereas the central ring is almost normal to them (5.3 (1)° for A/B, 81.8 (1)° for A/C and 87.0 (1)° for B/C). Comparing these values with those in the similar structure where the methyl subtitutent is in the para position (5.1° for A/B, 86.28° for A/C and 84.19° for B/C), there are no noticeable differences (Au & Tafeenko, 1986).

In the crystal structure, the molecular packing is made up of a network of weak hydrogen-bonding interactions (Fig. 2 & Table 1), favouring the formation of centrosymmetric dimers. Such conformations bring the C═O and N—H bonds into a syn orientation. The intermolecular distance between the centroids of the parallel benzene rings is ca. 3.77 Å. This value suggests the absence of any relevant π-stacking interactions.

### Experimental

0.0235 mol benzoin, 0.0235 mol 3-methylaniline and 0.0235 mol boric acid were added to 10 ml of ethyleneglycol. The mixture was heated to reflux for 1 h, then 15 ml of ethanol were added and the mixture cooled to RT. The reaction was followed using TLC. The yellow precipitate obtained was washed with cold water and ethanol (yield 85%). Yellow needle-like crystals, suitable for x-ray diffraction analysis, were obtained after a week by slow evaporation of a solution in ethanol.

### Refinement

The NH H-atom was located in difference electron-density map and was freely refined: N-H = 0.858 (17) Å. The C-bound H-atoms were included in calculated positions and treated as riding atoms: C-H = 0.98 Å, 0.93 Å and 0.96 Å for tertiary CH, aromatic CH and CH_3_ H-atoms, respectively, with U_iso_(H) = k × U_eq_(C), where k = 1.2 for CH H-atoms, and 1.5 for CH_3_ H-atoms.

### Crystal data

**Table:**

C_21_H_19_NO	*Z* = 2
*M**_r_* = 301.37	*F*(000) = 320
Triclinic, *P*1	*D*_x_ = 1.242 Mg m^−^^3^
Hall symbol: -P 1	Melting point: 385.14 K
*a* = 6.0510 (3) Å	Cu *K*α radiation, λ = 1.54184 Å
*b* = 11.5745 (4) Å	Cell parameters from 4346 reflections
*c* = 12.9458 (7) Å	θ = 3.8–66.7°
α = 112.542 (5)°	µ = 0.59 mm^−^^1^
β = 97.396 (4)°	*T* = 293 K
γ = 99.960 (4)°	Needle, yellow
*V* = 805.62 (8) Å^3^	0.34 × 0.12 × 0.07 mm

### Data collection

**Table:**

Oxford Diffraction Xcalibur Gemini S diffractometer	2833 independent reflections
Radiation source: Enhance (Cu) X-ray Source	2174 reflections with *I* > 2σ(*I*)
graphite	*R*_int_ = 0.027
Detector resolution: 16.0827 pixels mm^-1^	θ_max_ = 66.7°, θ_min_ = 3.8°
ω scans	*h* = −6→7
Absorption correction: part of the refinement model (Δ*F*) [cubic fit to sin(theta)/lambda - 24 parameters; Parkin *et al.* (1995)]	*k* = −10→13
*T*_min_ = 0.919, *T*_max_ = 0.960	*l* = −15→14
8027 measured reflections	

### Refinement

**Table:**

Refinement on *F*^2^	Secondary atom site location: difference Fourier map
Least-squares matrix: full	Hydrogen site location: inferred from neighbouring sites
*R*[*F*^2^ > 2σ(*F*^2^)] = 0.043	H atoms treated by a mixture of independent and constrained refinement
*wR*(*F*^2^) = 0.137	*w* = 1/[σ^2^(*F*_o_^2^) + (0.0863*P*)^2^] where *P* = (*F*_o_^2^ + 2*F*_c_^2^)/3
*S* = 1.09	(Δ/σ)_max_ < 0.001
2833 reflections	Δρ_max_ = 0.25 e Å^−^^3^
213 parameters	Δρ_min_ = −0.16 e Å^−^^3^
0 restraints	Extinction correction: *SHELXL97* (Sheldrick, 2008), Fc^*^=kFc[1+0.001xFc^2^λ^3^/sin(2θ)]^-1/4^
Primary atom site location: structure-invariant direct methods	Extinction coefficient: 0.0041 (11)

### Special details

**Table:**

Experimental. Refinement of F^2^ against ALL reflections. The weighted R-factor wR and goodness of fit S are based on F^2^, conventional R-factors R are based on F, with F set to zero for negative F^2^. The threshold expression of F^2^ > 2sigma(F^2^) is used only for calculating R-factors(gt) etc. and is not relevant to the choice of reflections for refinement. R-factors based on F^2^ are statistically about twice as large as those based on F, and R- factors based on ALL data will be even larger.
Geometry. Bond distances, angles etc. have been calculated using the rounded fractional coordinates. All su's are estimated from the variances of the (full) variance-covariance matrix. The cell esds are taken into account in the estimation of distances, angles and torsion angles
Refinement. Refinement of *F*^2^ against ALL reflections. The weighted *R*-factor wR and goodness of fit *S* are based on *F*^2^, conventional *R*-factors *R* are based on *F*, with *F* set to zero for negative *F*^2^. The threshold expression of *F*^2^ > σ(*F*^2^) is used only for calculating *R*-factors(gt) etc. and is not relevant to the choice of reflections for refinement. *R*-factors based on *F*^2^ are statistically about twice as large as those based on *F*, and *R*- factors based on ALL data will be even larger.

### Fractional atomic coordinates and isotropic or equivalent isotropic displacement parameters (Å^2^)

**Table:**

	*x*	*y*	*z*	*U*_iso_*/*U*_eq_	
O1	0.52659 (18)	0.50924 (11)	0.14596 (9)	0.0662 (4)	
N1	0.1300 (2)	0.38301 (13)	0.00199 (11)	0.0529 (4)	
C1	0.6200 (3)	0.63202 (19)	0.38272 (15)	0.0714 (6)	
C2	0.6620 (4)	0.6946 (2)	0.50033 (17)	0.0880 (8)	
C3	0.4898 (4)	0.6891 (2)	0.55688 (17)	0.0847 (8)	
C4	0.2717 (4)	0.6232 (2)	0.49658 (17)	0.0863 (8)	
C5	0.2260 (3)	0.56261 (18)	0.37817 (15)	0.0707 (6)	
C6	0.4006 (3)	0.56467 (14)	0.31949 (13)	0.0497 (5)	
C7	0.3671 (2)	0.49865 (14)	0.19224 (13)	0.0480 (5)	
C8	0.1322 (2)	0.41085 (13)	0.12058 (12)	0.0452 (4)	
C9	0.0958 (2)	0.28933 (13)	0.14383 (11)	0.0454 (4)	
C10	−0.0802 (3)	0.25707 (16)	0.19337 (14)	0.0586 (5)	
C11	−0.1102 (3)	0.14576 (19)	0.21171 (17)	0.0757 (7)	
C12	0.0364 (4)	0.06604 (19)	0.18093 (18)	0.0819 (7)	
C13	0.2102 (3)	0.09623 (17)	0.13051 (17)	0.0742 (7)	
C14	0.2411 (3)	0.20731 (15)	0.11226 (14)	0.0574 (5)	
C15	−0.0560 (2)	0.30252 (13)	−0.08485 (12)	0.0452 (4)	
C16	−0.2728 (3)	0.26933 (15)	−0.06433 (14)	0.0540 (5)	
C17	−0.4522 (3)	0.18600 (16)	−0.15360 (14)	0.0597 (6)	
C18	−0.4218 (3)	0.13398 (16)	−0.26408 (15)	0.0633 (6)	
C19	−0.2086 (3)	0.16684 (16)	−0.28761 (14)	0.0578 (5)	
C20	−0.0286 (3)	0.25155 (14)	−0.19799 (13)	0.0510 (5)	
C21	−0.1709 (4)	0.1084 (2)	−0.40766 (16)	0.0896 (8)	
H1	0.74080	0.63510	0.34540	0.0860*	
H2	0.81000	0.74100	0.54130	0.1060*	
H3	0.52000	0.73000	0.63640	0.1020*	
H4	0.15330	0.61900	0.53520	0.1040*	
H5	0.07610	0.52010	0.33780	0.0850*	
H8	0.01180	0.45580	0.14410	0.0540*	
H10	−0.17960	0.31080	0.21460	0.0700*	
H11	−0.22970	0.12480	0.24490	0.0910*	
H12	0.01770	−0.00810	0.19430	0.0980*	
H13	0.30790	0.04160	0.10850	0.0890*	
H14	0.36020	0.22730	0.07850	0.0690*	
H16	−0.29700	0.30340	0.00980	0.0650*	
H17	−0.59640	0.16470	−0.13870	0.0720*	
H18	−0.54410	0.07680	−0.32300	0.0760*	
H20	0.11400	0.27500	−0.21360	0.0610*	
H21A	−0.31250	0.05270	−0.45850	0.1340*	
H21B	−0.05730	0.05940	−0.40990	0.1340*	
H21C	−0.11860	0.17570	−0.43120	0.1340*	
H22	0.262 (3)	0.4037 (17)	−0.0130 (15)	0.062 (5)*	

### Atomic displacement parameters (Å^2^)

**Table:**

	*U*^11^	*U*^22^	*U*^33^	*U*^12^	*U*^13^	*U*^23^
O1	0.0533 (7)	0.0727 (8)	0.0548 (7)	−0.0058 (6)	0.0118 (5)	0.0167 (6)
N1	0.0473 (7)	0.0602 (8)	0.0438 (7)	0.0004 (6)	0.0051 (6)	0.0207 (6)
C1	0.0584 (10)	0.0864 (13)	0.0546 (10)	0.0023 (9)	0.0014 (8)	0.0235 (9)
C2	0.0714 (12)	0.1108 (17)	0.0540 (11)	−0.0008 (11)	−0.0099 (10)	0.0220 (11)
C3	0.1014 (16)	0.0868 (14)	0.0459 (10)	0.0088 (12)	0.0028 (10)	0.0164 (10)
C4	0.0896 (14)	0.0886 (14)	0.0559 (11)	0.0028 (12)	0.0230 (10)	0.0096 (10)
C5	0.0637 (10)	0.0698 (11)	0.0536 (10)	0.0000 (9)	0.0116 (8)	0.0066 (9)
C6	0.0528 (8)	0.0442 (8)	0.0467 (8)	0.0051 (6)	0.0043 (7)	0.0180 (7)
C7	0.0482 (8)	0.0424 (8)	0.0495 (9)	0.0043 (6)	0.0072 (7)	0.0189 (7)
C8	0.0449 (7)	0.0435 (7)	0.0419 (8)	0.0067 (6)	0.0063 (6)	0.0148 (6)
C9	0.0449 (7)	0.0429 (8)	0.0373 (7)	0.0024 (6)	−0.0008 (6)	0.0114 (6)
C10	0.0547 (9)	0.0597 (9)	0.0553 (10)	0.0018 (7)	0.0080 (7)	0.0239 (8)
C11	0.0766 (12)	0.0717 (12)	0.0716 (13)	−0.0114 (10)	0.0029 (10)	0.0387 (10)
C12	0.0939 (14)	0.0551 (10)	0.0810 (14)	−0.0101 (10)	−0.0212 (11)	0.0360 (10)
C13	0.0802 (12)	0.0503 (10)	0.0758 (12)	0.0156 (9)	−0.0107 (10)	0.0177 (9)
C14	0.0583 (9)	0.0513 (9)	0.0527 (9)	0.0100 (7)	0.0032 (7)	0.0152 (7)
C15	0.0466 (8)	0.0422 (7)	0.0455 (8)	0.0090 (6)	0.0030 (6)	0.0199 (6)
C16	0.0487 (8)	0.0590 (9)	0.0502 (9)	0.0106 (7)	0.0078 (7)	0.0202 (7)
C17	0.0452 (8)	0.0632 (10)	0.0640 (11)	0.0057 (7)	0.0031 (7)	0.0253 (9)
C18	0.0583 (10)	0.0554 (9)	0.0591 (10)	0.0013 (8)	−0.0093 (8)	0.0178 (8)
C19	0.0662 (10)	0.0531 (9)	0.0479 (9)	0.0111 (7)	0.0037 (7)	0.0187 (7)
C20	0.0531 (8)	0.0517 (8)	0.0481 (9)	0.0092 (7)	0.0085 (7)	0.0230 (7)
C21	0.0968 (15)	0.0917 (15)	0.0515 (11)	0.0013 (12)	0.0072 (10)	0.0113 (10)

### Geometric parameters (Å, °)

**Table:**

O1—C7	1.2123 (18)	C17—C18	1.374 (2)
N1—C8	1.4405 (19)	C18—C19	1.386 (3)
N1—C15	1.3810 (19)	C19—C20	1.388 (2)
N1—H22	0.862 (19)	C19—C21	1.505 (3)
C1—C6	1.383 (3)	C1—H1	0.9300
C1—C2	1.378 (3)	C2—H2	0.9300
C2—C3	1.355 (3)	C3—H3	0.9300
C3—C4	1.365 (3)	C4—H4	0.9300
C4—C5	1.385 (3)	C5—H5	0.9300
C5—C6	1.381 (3)	C8—H8	0.9800
C6—C7	1.495 (2)	C10—H10	0.9300
C7—C8	1.534 (2)	C11—H11	0.9300
C8—C9	1.534 (2)	C12—H12	0.9300
C9—C14	1.388 (2)	C13—H13	0.9300
C9—C10	1.381 (2)	C14—H14	0.9300
C10—C11	1.383 (3)	C16—H16	0.9300
C11—C12	1.375 (3)	C17—H17	0.9300
C12—C13	1.371 (3)	C18—H18	0.9300
C13—C14	1.380 (3)	C20—H20	0.9300
C15—C20	1.398 (2)	C21—H21A	0.9600
C15—C16	1.390 (2)	C21—H21B	0.9600
C16—C17	1.380 (2)	C21—H21C	0.9600
			
C8—N1—C15	122.36 (13)	C6—C1—H1	120.00
C8—N1—H22	115.3 (12)	C1—C2—H2	120.00
C15—N1—H22	120.7 (12)	C3—C2—H2	120.00
C2—C1—C6	120.89 (18)	C2—C3—H3	120.00
C1—C2—C3	120.6 (2)	C4—C3—H3	120.00
C2—C3—C4	119.66 (19)	C3—C4—H4	120.00
C3—C4—C5	120.3 (2)	C5—C4—H4	120.00
C4—C5—C6	120.71 (18)	C4—C5—H5	120.00
C1—C6—C7	118.15 (15)	C6—C5—H5	120.00
C1—C6—C5	117.76 (15)	N1—C8—H8	109.00
C5—C6—C7	124.08 (15)	C7—C8—H8	109.00
O1—C7—C8	119.84 (13)	C9—C8—H8	109.00
O1—C7—C6	120.53 (14)	C9—C10—H10	120.00
C6—C7—C8	119.55 (13)	C11—C10—H10	120.00
N1—C8—C9	112.77 (13)	C10—C11—H11	120.00
C7—C8—C9	107.94 (12)	C12—C11—H11	120.00
N1—C8—C7	108.31 (12)	C11—C12—H12	120.00
C10—C9—C14	118.55 (16)	C13—C12—H12	120.00
C8—C9—C10	122.18 (13)	C12—C13—H13	120.00
C8—C9—C14	119.26 (13)	C14—C13—H13	120.00
C9—C10—C11	120.72 (17)	C9—C14—H14	120.00
C10—C11—C12	120.06 (19)	C13—C14—H14	120.00
C11—C12—C13	119.8 (2)	C15—C16—H16	120.00
C12—C13—C14	120.36 (19)	C17—C16—H16	120.00
C9—C14—C13	120.52 (16)	C16—C17—H17	119.00
C16—C15—C20	117.97 (14)	C18—C17—H17	119.00
N1—C15—C16	122.36 (13)	C17—C18—H18	120.00
N1—C15—C20	119.68 (13)	C19—C18—H18	120.00
C15—C16—C17	120.25 (15)	C15—C20—H20	119.00
C16—C17—C18	121.25 (17)	C19—C20—H20	119.00
C17—C18—C19	119.89 (17)	C19—C21—H21A	109.00
C18—C19—C20	118.88 (16)	C19—C21—H21B	109.00
C18—C19—C21	120.58 (17)	C19—C21—H21C	110.00
C20—C19—C21	120.51 (17)	H21A—C21—H21B	109.00
C15—C20—C19	121.73 (16)	H21A—C21—H21C	110.00
C2—C1—H1	120.00	H21B—C21—H21C	109.00
			
C15—N1—C8—C7	177.87 (14)	N1—C8—C9—C14	55.51 (17)
C15—N1—C8—C9	58.48 (18)	C7—C8—C9—C10	117.19 (14)
C8—N1—C15—C16	17.0 (2)	C7—C8—C9—C14	−64.09 (16)
C8—N1—C15—C20	−162.92 (15)	C8—C9—C10—C11	179.09 (15)
C6—C1—C2—C3	1.5 (4)	C14—C9—C10—C11	0.4 (2)
C2—C1—C6—C5	0.1 (3)	C8—C9—C14—C13	−178.99 (15)
C2—C1—C6—C7	−179.82 (19)	C10—C9—C14—C13	−0.2 (2)
C1—C2—C3—C4	−1.4 (4)	C9—C10—C11—C12	0.2 (3)
C2—C3—C4—C5	−0.3 (4)	C10—C11—C12—C13	−1.0 (3)
C3—C4—C5—C6	1.9 (4)	C11—C12—C13—C14	1.1 (3)
C4—C5—C6—C1	−1.8 (3)	C12—C13—C14—C9	−0.5 (3)
C4—C5—C6—C7	178.17 (19)	N1—C15—C16—C17	−178.40 (17)
C1—C6—C7—O1	−3.4 (3)	C20—C15—C16—C17	1.5 (3)
C1—C6—C7—C8	173.22 (17)	N1—C15—C20—C19	177.80 (17)
C5—C6—C7—O1	176.72 (18)	C16—C15—C20—C19	−2.2 (3)
C5—C6—C7—C8	−6.7 (3)	C15—C16—C17—C18	0.1 (3)
O1—C7—C8—N1	−16.0 (2)	C16—C17—C18—C19	−1.1 (3)
O1—C7—C8—C9	106.37 (17)	C17—C18—C19—C20	0.5 (3)
C6—C7—C8—N1	167.39 (14)	C17—C18—C19—C21	178.57 (19)
C6—C7—C8—C9	−70.22 (17)	C18—C19—C20—C15	1.1 (3)
N1—C8—C9—C10	−123.21 (14)	C21—C19—C20—C15	−176.92 (18)

### Hydrogen-bond geometry (Å, °)

**Table:**

*D*—H···*A*	*D*—H	H···*A*	*D*···*A*	*D*—H···*A*
N1—H22···O1^i^	0.859 (17)	2.660 (17)	3.3913 (17)	143.8 (15)

Symmetry codes: (i) −*x*+1, −*y*+1, −*z*.
